# Associations of derived inflammatory, lipid, and anthropometric indices with CT-detected pulmonary nodules: a hospital-based cross-sectional study with explainable machine learning

**DOI:** 10.3389/fendo.2026.1922394

**Published:** 2026-09-01

**Authors:** Mengmeng Wang, Yanyan Zhou, Beibei Wu, Yuan Liu, Xiao Luo, Anan Zhang

**Affiliations:** 1 Xiyuan Hospital, China Academy of Chinese Medical Sciences, Beijing, China; 2 The Second Affiliated Hospital, Shandong First Medical University and Shandong Academy of Medical Sciences, Taian, China; 3 Chongqing University Fuling Hospital, Chongqing University, Chongqing, China; 4 School of Nursing and Rehabilitation, North China University of Science and Technology, Tangshan, China

**Keywords:** atherogenic index of plasma, explainable machine learning, pulmonary nodule, systemic inflammation, systemic inflammation response index, systems endocrinology

## Abstract

**Background:**

Pulmonary nodules are frequently detected during routine health-examination chest computed tomography (CT). From a systems-endocrinology perspective, routinely available indices integrating adiposity distribution, lipid metabolism, immune-cell balance, and low-grade inflammation may characterize cross-organ metabolic-inflammatory phenotypes, but their associations with CT-detected pulmonary nodules remain uncertain.

**Methods:**

This hospital-based cross-sectional study included 1,072 adults undergoing routine health examinations at the Second Affiliated Hospital of Shandong First Medical University from 2024 to 2025. The primary outcome was any CT-detected pulmonary nodule; the secondary outcome was an institutional Lung-RADS category ≥3. Right-skewed indices were natural-log transformed before standardization. We applied multivariable logistic regression, Benjamini-Hochberg false-discovery-rate (FDR) correction, a reduced non-redundant representative-index model, restricted cubic splines, repeated nested cross-validation, and machine-learning models with SHAP explanations computed only for held-out observations.

**Results:**

CT-detected pulmonary nodules were present in 381 participants (35.54%), and 109 participants (10.17%) had Lung-RADS category ≥3. In the mutually adjusted representative-index model, AIP (OR, 1.38; 95% CI, 1.16-1.64; q = 0.003) and SIRI (OR, 1.37; 95% CI, 1.14-1.64; q = 0.004) were associated with any pulmonary nodule. For the exploratory secondary Lung-RADS category ≥3 outcome (109 events; 6.41 events per model parameter), SIRI was associated with Lung-RADS category ≥3 (OR, 1.48; 95% CI, 1.15-1.91; q = 0.008), but this low-EPV estimate should be interpreted cautiously. In exploratory single-index analyses, several lipid-adiposity and monocyte-related indices remained significant after FDR correction. The basic risk model achieved a cross-validated AUC of 0.76; the derived-index model achieved an AUC of 0.77, without a statistically significant AUC increment (difference, 0.01; P = 0.100).

**Conclusions:**

AIP and SIRI were independently associated with CT-detected pulmonary nodules. An exploratory association between SIRI and Lung-RADS category ≥3 was also observed; however, the limited number of events and EPV of 6.41 require cautious interpretation and independent validation. These cross-sectional findings describe an exploratory metabolic-inflammatory phenotype rather than a causal mechanism or clinically ready diagnostic tool. Multicenter longitudinal validation with standardized radiological characterization is required.

## 1 Introduction

Pulmonary nodules are among the most common incidental and screening-detected findings on chest CT. Large randomized trials have established the mortality benefit of low-dose CT in high-risk populations, but CT is also used in routine health examinations, where the population may be broader, younger, and not limited to heavy smokers (1, 2). This expansion has motivated investigation of readily available clinical phenotypes associated with nodule detection; however, such phenotypes cannot replace radiological characterization after a nodule is identified.

Guideline-based nodule management relies predominantly on radiological characteristics, including size, density, number, location, morphology, and interval growth (3, 4). Pulmonary nodules are biologically heterogeneous and may represent benign inflammatory lesions, granulomas, scars, indeterminate nodules, or early malignant lesions. Consequently, an analysis that groups all CT-detected nodules together should be interpreted as an exploratory assessment of an imaging phenotype rather than as an analysis of a single disease entity or a malignancy diagnosis. Population-based and hospital-based studies have nevertheless reported substantial nodule detection rates, particularly with increasing age, cumulative smoking exposure, and broader CT use (5–11).

Inflammatory and metabolic profiles may be relevant to the biological context in which pulmonary nodules are detected. Chronic inflammatory activation, oxidative stress, myeloid-cell recruitment, adiposity, and dyslipidemia are interconnected systemic processes that may accompany tissue remodeling and tumor-promoting microenvironments (12–14). In Chinese populations, peripheral inflammatory markers have been associated with pulmonary nodule detection and incident lung cancer, supporting further evaluation of immune-inflammatory profiles while not establishing causality (9).

Composite indices derived from routine health-examination variables may summarize information that single markers do not. Ratios and composite scores integrating neutrophils, lymphocytes, monocytes, platelets, HDL cholesterol, triglycerides, albumin, and anthropometric measures have been proposed as accessible markers of systemic inflammation, atherogenic dyslipidemia, visceral adiposity, and immune-nutritional status (15–32). We selected candidate indices *a priori* according to these biological domains and the availability of their component variables. Because several indices are mathematically nested or share the same components, they were not assumed to represent distinct independent mechanisms.

From a systems-endocrinology perspective, adipose distribution, lipid handling, glycemic regulation, and immune-inflammatory signaling are coordinated processes that may be reflected across organs. In the present study, pulmonary nodule status was treated as an extra-endocrine imaging phenotype through which cross-organ metabolic-inflammatory associations could be examined; it was not treated as an endocrine disease, a surrogate diagnosis of lung cancer, or a basis for replacing radiological assessment.

Machine-learning approaches may detect nonlinear and interactive patterns across correlated biomarkers, but their use in medical research requires transparent reporting, calibration assessment, out-of-sample evaluation, and interpretability. TRIPOD and TRIPOD+AI emphasize complete reporting of model development and validation, whereas SHAP explanations provide a framework for interpreting tree-based predictions (33–36). Whether routine health-examination-derived indices add reproducible predictive information beyond conventional risk factors remains uncertain.

We therefore conducted a hospital-based cross-sectional study using routine health-examination data and brief questionnaire information from the Health Examination Center of the Second Affiliated Hospital of Shandong First Medical University. We evaluated associations of inflammatory, lipid, nutritional, and anthropometric indices with any CT-detected pulmonary nodule and with Lung-RADS category ≥3; used a reduced non-redundant set of representative indices as the primary multivariable analysis; and compared regularized regression with interpretable machine-learning models for exploratory risk phenotyping.

## 2 Materials and methods

### 2.1 Study design, setting, and ethics

This hospital-based cross-sectional study included individuals who underwent routine health examinations at the Health Examination Center of the Second Affiliated Hospital of Shandong First Medical University between June 2024 and December 2025. The analytic file combined routine health-examination data with brief questionnaire information. All participants provided written informed consent. The report was prepared according to STROBE and TRIPOD principles for observational and prediction-model studies (33, 37).

### 2.2 Outcome assessment

All participants underwent standard-dose chest CT. Each examination was initially interpreted by one radiologist with an intermediate professional title or above, and the report underwent review by a second radiologist. Laboratory results were not available to the radiologists during image interpretation, and nodule count was entered by the radiologists. The primary outcome was any CT-detected pulmonary nodule. The secondary outcome was a binary institutional database field coded as positive when the reviewed radiology report assigned Lung-RADS category ≥3 (4). Lung-RADS categorization was not applied routinely to all health-examination participants. When categorization was requested by a patient or recommended by a physician according to the clinical context, ACR Lung-RADS v2022 was used as the institutional reporting framework. The initial interpreting radiologist assigned the Lung-RADS category, which was reviewed by a second radiologist; all radiologists involved in these examinations had received standardized training in the institutional reporting protocol. Because the cohort was not defined by guideline-based lung-cancer screening eligibility, Lung-RADS category ≥3 was treated as an institutional secondary imaging-stratification outcome rather than as a screening-eligibility or malignancy endpoint. Nodule-count category was used only for descriptive characterization and was not entered as a predictor, thereby avoiding outcome-derived information leakage. The database did not contain retrievable standardized information on nodule size, morphology, density, location, growth, or follow-up evolution, and centralized image re-reading was not performed.

### 2.3 Clinical, questionnaire, and laboratory variables

Variables available for analysis included age, sex, height, weight, waist circumference, hip circumference, systolic and diastolic blood pressure, smoking status, current alcohol drinking, regular exercise, complete blood count parameters, albumin, lipid profile, fasting plasma glucose, liver enzymes, creatinine, estimated glomerular filtration rate (eGFR), uric acid, hsCRP, pulmonary nodule status, Lung-RADS category ≥3 status, and nodule-count category. Central obesity was defined as waist circumference ≥90 cm in men or ≥80 cm in women (38). The examination-defined hypertension variable was coded as systolic blood pressure ≥140 mmHg or diastolic blood pressure ≥90 mmHg at the single health-examination measurement (39); prior hypertension diagnoses and antihypertensive medication use were not incorporated. Detailed smoking pack-years, time since cessation, occupational or environmental exposures, family history of lung cancer, and detailed chronic lung-disease history were not available.

### 2.4 Construction of derived indices

Derived anthropometric, lipid, inflammatory, and nutritional indices were recalculated from raw variables to ensure analytic reproducibility. Anthropometric indices included BMI = weight (kg)/height (m)^2, WHtR = waist circumference/height, WHR = waist circumference/hip circumference, BRI = 364.2 - 365.5 x sqrt[1 - {(waist circumference/2π)^2/(0.5 x height)^2}], and WWI = waist circumference/sqrt[weight (kg)] (23, 24, 26–28). Lipid-related indices included non-HDL-C = total cholesterol - HDL-C, remnant cholesterol = total cholesterol - LDL-C - HDL-C, and AIP = log10(triglycerides/HDL-C) (21, 29, 30). CMI was calculated as (triglycerides/HDL-C) x WHtR (22). Inflammatory indices included NLR = neutrophil count/lymphocyte count, PLR = platelet count/lymphocyte count, MLR = monocyte count/lymphocyte count, SII = platelet count x neutrophil count/lymphocyte count, SIRI = neutrophil count x monocyte count/lymphocyte count, AISI = neutrophil count x monocyte count x platelet count/lymphocyte count, and MHR = monocyte count/HDL-C (15–20). Nutritional-inflammatory indices included PNI = albumin + 5 x lymphocyte count and HALP = hemoglobin x albumin x lymphocyte count/platelet count (31, 32).

The candidate indices were prespecified on the basis of published formulas, biological-domain coverage, and calculability from routine variables rather than selected according to their associations with the outcomes. To address mathematical nesting and redundancy, we examined the absolute Spearman correlation structure and used outcome-independent hierarchical grouping, with |rho| ≥0.80 indicating a highly correlated cluster. One interpretable representative was retained from each major domain for the primary simultaneous model: WHtR (anthropometry), AIP (lipid balance), SIRI (cell-based inflammation), hsCRP (acute-phase inflammation), and PNI (immune-nutrition). Variance inflation factors (VIFs) were calculated for the full 17-parameter representative-index design matrix, including the five representative indices and all adjustment covariates (with categorical variables represented by indicator terms), rather than only among the five representative indices. The full list of indices was retained only for FDR-controlled exploratory single-index analyses.

For predictive modeling of any pulmonary nodule, four feature sets were defined using unique variables. The basic risk model contained 11 variables: age, sex, smoking status, current alcohol drinking, regular exercise, central obesity, examination-defined hypertension, BMI, systolic blood pressure, diastolic blood pressure, and fasting plasma glucose. The traditional raw model contained 33 unique variables, comprising the basic variables and available raw anthropometric and laboratory measurements. The derived-index model contained 29 unique variables, comprising the basic variables, 17 non-BMI derived indices, and hsCRP. The combined model contained the union of the raw and derived sets (50 unique variables). The traditional and derived sets shared 12 variables; therefore, 33 + 29 - 12 = 50 unique variables in the combined model. For association analyses, eGFR was selected as the renal-function adjustment variable and creatinine was not entered simultaneously to avoid redundant adjustment for overlapping renal-function information. In contrast, the prediction feature sets were designed to evaluate routinely available candidate predictors; therefore, creatinine and eGFR were both retained as separate raw predictors in the traditional raw and combined feature sets, with their correlation handled by penalization and/or model constraints.

### 2.5 Statistical analysis

Continuous variables are presented as mean (SD), and categorical variables as number (percentage). Between-group comparisons used Welch t tests for continuous variables and chi-square tests for categorical variables. Before association modeling, skewness was calculated without reference to outcome status. Positive variables with skewness >1.0 were natural-log transformed and then standardized; this rule resulted in log transformation of CMI, AISI, and hsCRP. AIP was retained in its published log10(triglycerides/HDL-C) form. All other examined indices were standardized on their original scale. Distributional diagnostics and transformations are reported in **Supplementary Table 4**.

The primary association analysis simultaneously included the five representative indices (WHtR, AIP, SIRI, hsCRP, and PNI) and adjusted for age, sex, smoking status, current alcohol drinking, regular exercise, examination-defined hypertension, fasting plasma glucose, uric acid, eGFR, ALT, and GGT. Because WHtR was included as the representative adiposity measure, BMI was not additionally included in this joint model. VIF <5 was considered evidence against problematic residual multicollinearity. In secondary exploratory analyses, each of 20 indices was modeled separately per 1-SD increment using the same covariate strategy; BMI was additionally included when the exposure was not adiposity-derived. Benjamini-Hochberg FDR correction was applied jointly across the 40 single-index tests (20 indices x 2 outcomes), and both nominal P values and q values were reported (40). The Lung-RADS category ≥3 association analysis was considered exploratory because only 109 events were available, corresponding to 6.41 events per model parameter in the 17-parameter representative-index model; these secondary estimates were therefore interpreted cautiously.

Restricted cubic spline analyses were performed for the four representative markers with an overall association or specific biological relevance (WHtR, AIP, SIRI, and hsCRP). Spline plots display adjusted ORs and 95% CIs relative to the median exposure value; x-axis ranges were restricted to the 1st to 99th percentiles. Overall and nonlinear components were tested separately. Prespecified subgroup analyses of SIRI, AIP, and WHtR were conducted by sex, age (<50 or ≥50 years), central obesity, and examination-defined hypertension, with interaction P values obtained from cross-product terms.

Prediction modeling was restricted to the primary outcome of any pulmonary nodule; no high-dimensional prediction model was fitted for Lung-RADS category ≥3 because 109 events would provide only 2.18 events per variable for the 50-feature combined model. For feature-set comparisons, ridge logistic regression was evaluated using repeated nested stratified cross-validation (five outer folds repeated three times, with three-fold inner tuning of the penalty parameter). Each participant therefore received only out-of-fold predictions, averaged across repeats. Model discrimination was summarized by AUC and AUPRC; calibration by calibration curves, calibration intercept, calibration slope, and Brier score; and clinical utility by decision-curve analysis. Participant-level bootstrap resampling (500 replicates) was used for 95% CIs and paired differences. Continuous NRI and IDI were reported only as exploratory secondary measures because these statistics may be unstable or inflated and should not override discrimination and calibration findings (41–43).

Machine-learning algorithms included ridge logistic regression, LASSO logistic regression, random forest, XGBoost, LightGBM, and gradient boosting. All algorithms used regularization and/or conservative complexity constraints and were evaluated from held-out predictions in stratified five-fold cross-validation. AUC differences were assessed by paired participant-level bootstrap resampling. The best-performing tree-based model was interpreted using SHAP values calculated exclusively for observations in the held-out outer fold; SHAP values were then pooled across folds to summarize global importance and feature-level contributions (34, 35). Analyses were performed using reproducible Python code with a fixed random seed (20260728), which is supplied with the revision. All tests were two-sided.

## 3 Results

### 3.1 Study population and outcome distribution

The analytic cohort included 1,072 participants. CT-detected pulmonary nodules were present in 381 participants (35.54%), and 109 participants (10.17%) had an institutional Lung-RADS category ≥3. Among participants with pulmonary nodules, 212 (55.64%) had a single nodule and 169 (44.36%) had multiple nodules. Baseline characteristics according to Lung-RADS category ≥3 status are shown in **Supplementary Table 1**.

### 3.2 Baseline characteristics

**Figure 1:**
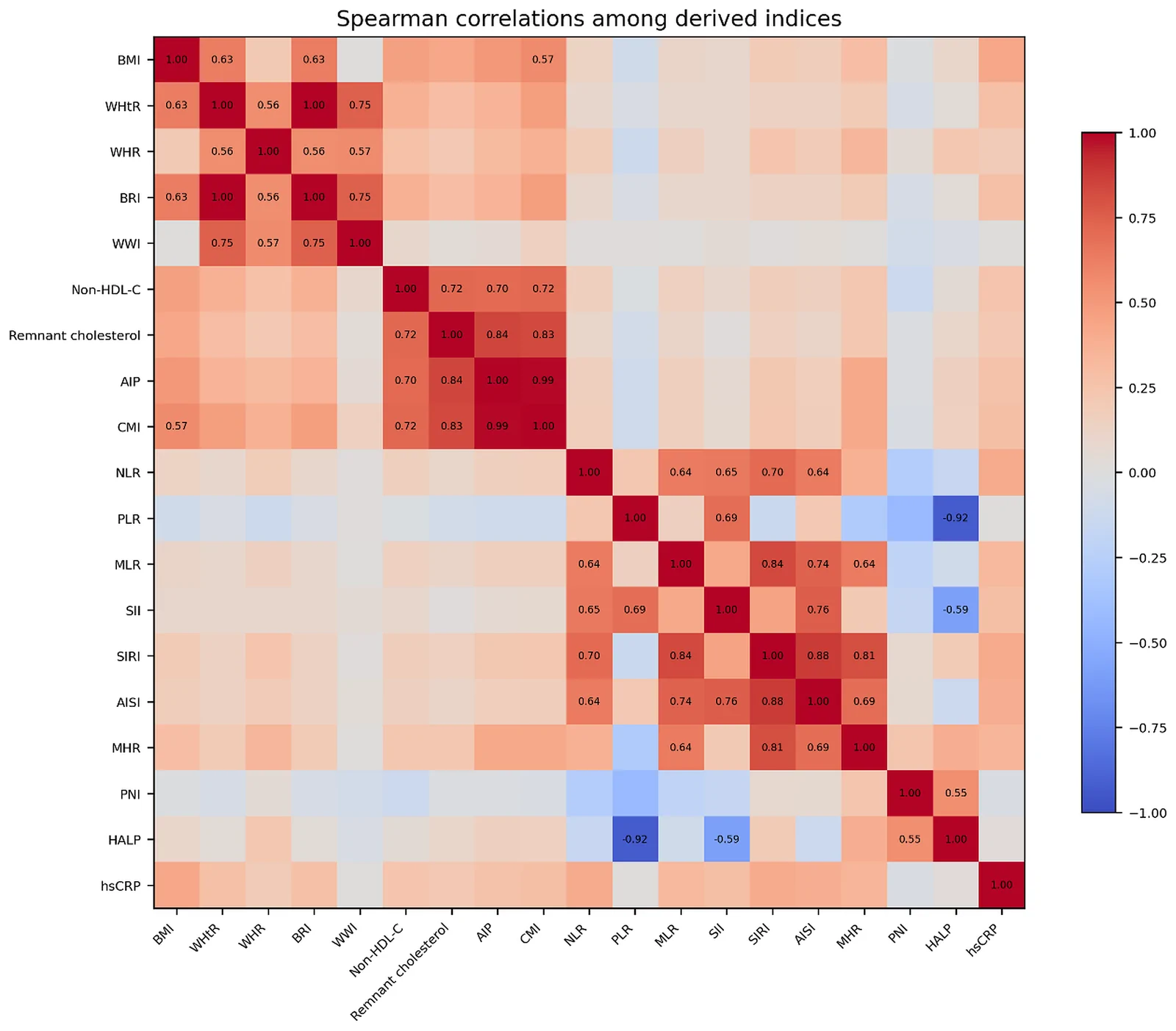
Spearman correlation heatmap of derived inflammatory, lipid, nutritional, and anthropometric indices. Variables are ordered by hierarchical clustering; numerical coefficients are displayed for absolute correlations ≥0.80, emphasizing mathematically nested and highly redundant indices.

Baseline characteristics according to pulmonary nodule status are shown in **Table 1**. Participants with pulmonary nodules were older than those without nodules (54.95 [11.89] vs 45.26 [11.73] years; P <0.001) and had higher mean waist circumference, blood pressure, several myeloid-cell measures, triglycerides, uric acid, and hsCRP. **Figure 1** demonstrates the expected redundancy among derived indices, including near-identity between WHtR and BRI and very high correlation between AIP and CMI. When VIFs were recalculated across the full 17-parameter representative-index design matrix, including both representative indices and adjustment covariates, values ranged from 1.02 to 2.62; all were below the prespecified threshold of 5, providing no evidence of problematic residual multicollinearity in the fitted design matrix.

**Table 1:** Baseline characteristics according to CT-detected pulmonary nodule status.

Characteristic	Pulmonary nodule			
	Overall	No	Yes	P value
Overall	1072	691	381	
Age, years	48.71 (12.66)	45.26 (11.73)	54.95 (11.89)	<0.001
Height, cm	165.74 (7.92)	165.17 (8.01)	166.78 (7.66)	0.001
Weight, kg	66.83 (10.19)	65.27 (9.86)	69.68 (10.17)	<0.001
Waist circumference, cm	83.05 (7.27)	81.50 (7.01)	85.85 (6.88)	<0.001
Hip circumference, cm	97.27 (5.26)	96.82 (5.33)	98.10 (5.04)	<0.001
Systolic blood pressure, mmHg	120.40 (12.49)	118.18 (12.31)	124.41 (11.79)	<0.001
Diastolic blood pressure, mmHg	76.34 (7.91)	74.93 (7.90)	78.90 (7.28)	<0.001
White blood cell count, 10^9/L	6.26 (0.95)	6.14 (0.91)	6.48 (0.98)	<0.001
Neutrophil count, 10^9/L	3.68 (0.68)	3.58 (0.65)	3.87 (0.69)	<0.001
Lymphocyte count, 10^9/L	1.96 (0.34)	1.95 (0.33)	1.99 (0.34)	0.061
Monocyte count, 10^9/L	0.41 (0.10)	0.39 (0.10)	0.44 (0.11)	<0.001
Platelet count, 10^9/L	236.07 (41.06)	235.55 (41.74)	237.02 (39.85)	0.568
Hemoglobin, g/L	143.78 (12.83)	142.50 (12.84)	146.11 (12.49)	<0.001
Albumin, g/L	45.26 (1.98)	45.51 (1.90)	44.82 (2.04)	<0.001
Total cholesterol, mmol/L	5.10 (0.85)	4.93 (0.80)	5.40 (0.87)	<0.001
Triglycerides, mmol/L	1.68 (0.88)	1.51 (0.79)	1.98 (0.95)	<0.001
HDL-C, mmol/L	1.26 (0.21)	1.29 (0.21)	1.22 (0.21)	<0.001
LDL-C, mmol/L	2.91 (0.63)	2.79 (0.60)	3.12 (0.62)	<0.001
Fasting plasma glucose, mmol/L	5.31 (0.77)	5.21 (0.75)	5.48 (0.77)	<0.001
ALT, U/L	27.66 (10.66)	26.45 (10.17)	29.87 (11.16)	<0.001
AST, U/L	23.04 (6.45)	22.67 (6.05)	23.71 (7.06)	0.015
GGT, U/L	37.14 (21.50)	35.53 (20.62)	40.05 (22.75)	0.001
Creatinine, umol/L	71.34 (12.47)	69.13 (12.13)	75.36 (12.07)	<0.001
eGFR, mL/min/1.73 m^2	105.84 (11.20)	108.19 (10.77)	101.57 (10.71)	<0.001
Uric acid, umol/L	334.69 (67.68)	322.91 (66.77)	356.07 (64.07)	<0.001
hsCRP, mg/L	1.42 (1.19)	1.21 (0.94)	1.81 (1.46)	<0.001
BMI, kg/m^2	24.25 (2.66)	23.85 (2.60)	24.97 (2.62)	<0.001
WHtR	0.50 (0.04)	0.49 (0.04)	0.52 (0.04)	<0.001
WHR	0.85 (0.07)	0.84 (0.07)	0.88 (0.06)	<0.001
Non-HDL-C, mmol/L	3.84 (0.88)	3.65 (0.81)	4.18 (0.89)	<0.001
Remnant cholesterol, mmol/L	0.93 (0.43)	0.85 (0.40)	1.06 (0.46)	<0.001
AIP	0.08 (0.25)	0.02 (0.24)	0.17 (0.24)	<0.001
CMI	0.71 (0.47)	0.62 (0.39)	0.89 (0.54)	<0.001
WWI	10.20 (0.67)	10.13 (0.67)	10.33 (0.66)	<0.001
BRI	3.42 (0.81)	3.28 (0.78)	3.68 (0.80)	<0.001
NLR	1.89 (0.29)	1.85 (0.27)	1.97 (0.31)	<0.001
PLR	124.17 (32.52)	124.85 (33.26)	122.95 (31.14)	0.351
MLR	0.21 (0.05)	0.20 (0.05)	0.23 (0.05)	<0.001
SII	447.42 (106.61)	437.06 (103.99)	466.23 (108.83)	<0.001
SIRI	0.79 (0.27)	0.74 (0.24)	0.89 (0.30)	<0.001
AISI	187.18 (74.44)	174.66 (67.31)	209.89 (81.17)	<0.001
MHR	0.34 (0.12)	0.32 (0.10)	0.38 (0.12)	<0.001
PNI	55.08 (2.64)	55.25 (2.58)	54.76 (2.73)	0.004
HALP	56.15 (16.23)	55.76 (16.38)	56.86 (15.97)	0.287
Sex				<0.001
Male	596 (55.60)	335 (48.48)	261 (68.50)	
Female	476 (44.40)	356 (51.52)	120 (31.50)	
Smoking status				<0.001
Never	708 (66.04)	511 (73.95)	197 (51.71)	
Former	83 (7.74)	38 (5.50)	45 (11.81)	
Current	281 (26.21)	142 (20.55)	139 (36.48)	
Current alcohol drinking				0.027
No	790 (73.69)	525 (75.98)	265 (69.55)	
Yes	282 (26.31)	166 (24.02)	116 (30.45)	
Regular exercise				0.536
No	621 (57.93)	395 (57.16)	226 (59.32)	
Yes	451 (42.07)	296 (42.84)	155 (40.68)	
Central obesity				<0.001
No	702 (65.49)	489 (70.77)	213 (55.91)	
Yes	370 (34.51)	202 (29.23)	168 (44.09)	
Examination-defined hypertension				<0.001
No	967 (90.21)	646 (93.49)	321 (84.25)	
Yes	105 (9.79)	45 (6.51)	60 (15.75)	

Data are shown as mean (SD) or No. (%). For categorical variables, levels are shown as indented rows and the global P value is presented on the parent variable row. P values were calculated using Welch t tests or chi-square tests, as appropriate, and are formatted as <0.001 or to three decimal places. Central obesity was defined as waist circumference ≥90 cm in men or ≥80 cm in women. Examination-defined hypertension was based on a single measurement with systolic blood pressure ≥140 mmHg or diastolic blood pressure ≥90 mmHg.

### 3.3 Primary representative-index associations and exploratory single-index analyses

In the mutually adjusted representative-index model (**Table 2**), AIP (OR, 1.38; 95% CI, 1.16-1.64; P <0.001; q = 0.003) and SIRI (OR, 1.37; 95% CI, 1.14-1.64; P <0.001; q = 0.004) were independently associated with any pulmonary nodule. WHtR showed a nominal association (OR, 1.21; 95% CI, 1.02-1.43; P = 0.028) that did not remain significant after FDR correction (q = 0.070); hsCRP and PNI were not independently associated. Notably, WHtR was significant in the corresponding exploratory single-index analysis (OR, 1.32; 95% CI, 1.12-1.55; P <0.001; q = 0.004), demonstrating attenuation after simultaneous adjustment for the other representative domains. For Lung-RADS category ≥3, SIRI was the only representative index that remained significant after FDR correction (OR, 1.48; 95% CI, 1.15-1.91; P = 0.002; q = 0.008). The representative models contained 17 model parameters, corresponding to 22.41 events per parameter for any pulmonary nodule and 6.41 for Lung-RADS category ≥3; the low-EPV Lung-RADS estimate was therefore treated as exploratory and interpreted cautiously.

**Table 2:** Mutually adjusted associations of the reduced representative-index set with pulmonary nodule outcomes.

Representative index	Any pulmonary nodule OR (95% CI)	P value	FDR q value	Lung-RADS ≥3 OR (95% CI)	P value	FDR q value
WHtR	1.21 (1.02-1.43)	0.028	0.070	1.11 (0.86-1.43)	0.432	0.486
AIP	1.38 (1.16-1.64)	<0.001	0.003	1.11 (0.85-1.45)	0.438	0.486
SIRI	1.37 (1.14-1.64)	<0.001	0.004	1.48 (1.15-1.91)	0.002	0.008
hsCRP	1.17 (0.99-1.38)	0.073	0.147	1.06 (0.83-1.36)	0.622	0.622
PNI	0.90 (0.77-1.05)	0.175	0.251	0.83 (0.66-1.05)	0.125	0.208

ORs are per 1-SD increment and are mutually adjusted for the other four representative indices and for age, sex, smoking status, current alcohol drinking, regular exercise, examination-defined hypertension, fasting plasma glucose, uric acid, eGFR, ALT, and GGT. hsCRP was natural-log transformed before standardization. Benjamini-Hochberg q values were calculated across the 10 representative-index outcome tests. VIFs were calculated across the full 17-parameter design matrix and ranged from 1.02 to 2.62. Examination-defined hypertension was based on a single measurement with systolic blood pressure ≥140 mmHg or diastolic blood pressure ≥90 mmHg. Events per model parameter were 22.41 for any pulmonary nodule and 6.41 for Lung-RADS category ≥3.

In the FDR-controlled exploratory single-index analyses (**Supplementary Table 2**), log-transformed CMI showed the largest association with any pulmonary nodule (OR, 1.52; 95% CI, 1.27-1.82; q <0.001). AIP, SIRI, MLR, AISI, MHR, WHtR, BRI, waist circumference, BMI, remnant cholesterol, NLR, SII, and log-transformed hsCRP also remained significant after FDR correction. For Lung-RADS category ≥3, the associations that survived FDR correction were concentrated in monocyte-related indices: MLR (OR, 1.50; q = 0.004), SIRI (OR, 1.49; q = 0.005), MHR (OR, 1.49; q = 0.007), and log-transformed AISI (OR, 1.36; q = 0.045). Because these indices share components and formulas, these findings are presented as related exploratory signals rather than as evidence for multiple independent biomarkers.

### 3.4 Nonlinearity and subgroup patterns

**Figure 2:**
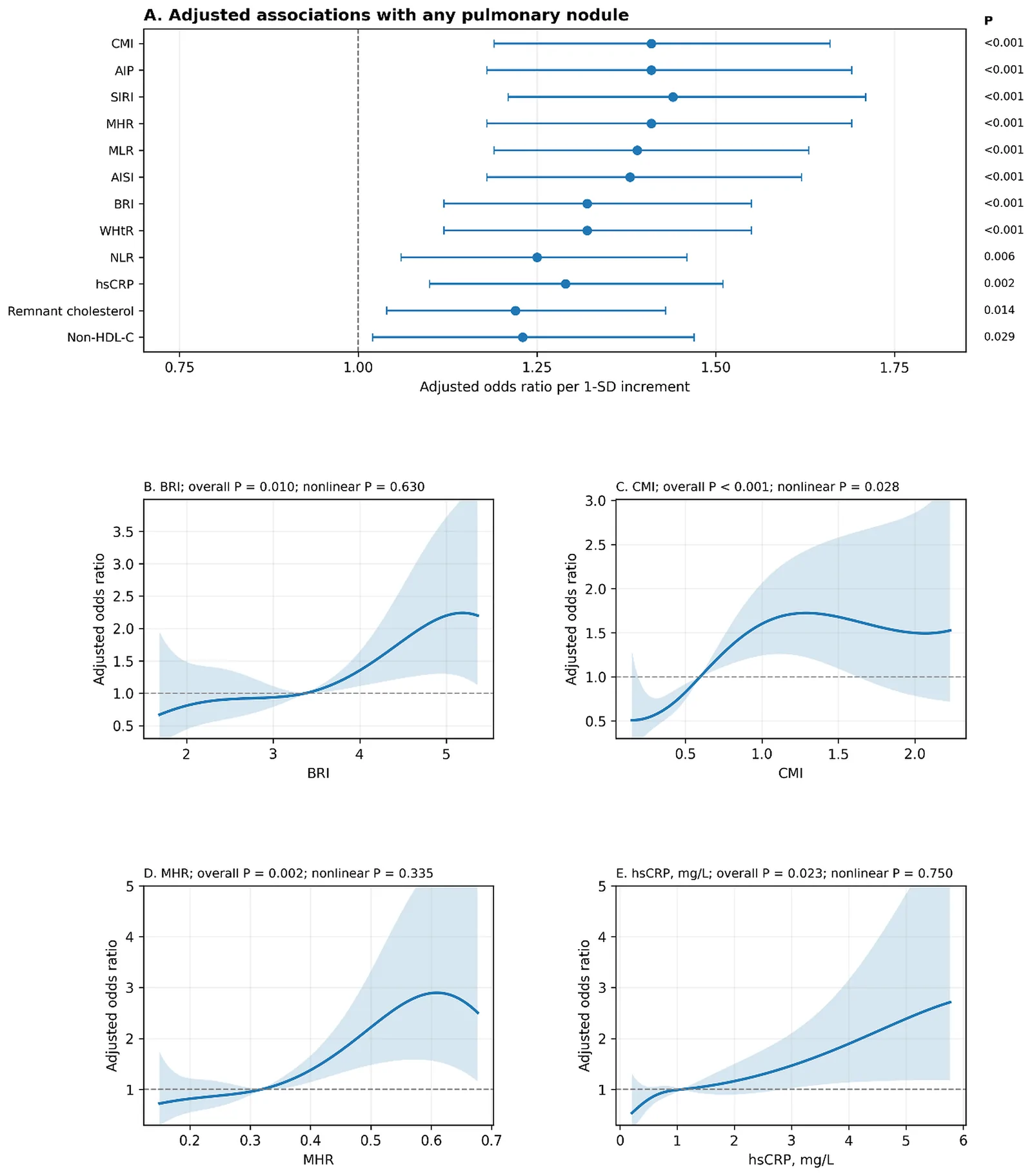
Mutually adjusted representative-index associations and restricted cubic spline assessment. **(A)** shows adjusted ORs for the five representative indices entered simultaneously. **(B–E)** show adjusted spline curves for WHtR, AIP, SIRI, and hsCRP, with reference at the median and x-axes restricted to the 1st-99th percentiles. CMI, AISI, and hsCRP were natural-log transformed before standardization where applicable; the hsCRP spline is shown on its clinical scale.

Restricted cubic spline analyses (**Figure 2**) showed overall associations for WHtR (P = 0.017), AIP (P = 0.002), SIRI (P = 0.001), and hsCRP (P = 0.033). None showed evidence of departure from linearity within the 1st-99th percentile range (all P for nonlinearity ≥0.521). Subgroup results are summarized in **Supplementary Table 3**. The estimates were generally directionally similar across sex, age, central-obesity, and examination-defined-hypertension strata, although subgroup analyses were exploratory and some strata contained limited events.

### 3.5 Incremental prediction, calibration, and machine learning

**Figure 3:**
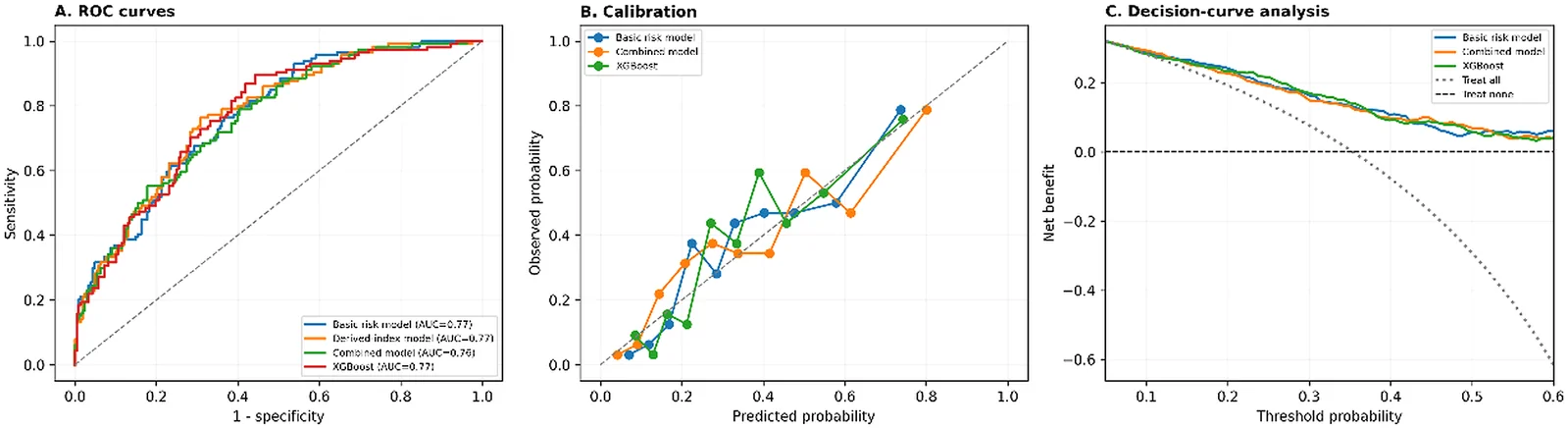
Repeated nested cross-validated prediction assessment for the four prespecified feature sets. **(A)** shows out-of-fold ROC curves; **(B)** shows out-of-fold calibration curves; **(C)** shows decision-curve analysis. The expanded models did not significantly improve AUC compared with the basic risk model.

Repeated nested cross-validation results are summarized in **Table 3** and **Figure 3**. The basic risk model achieved an AUC of 0.76 (95% CI, 0.72-0.78), AUPRC of 0.64, and Brier score of 0.19. The traditional raw, derived-index, and combined models achieved AUCs of 0.76, 0.77, and 0.76, respectively; none significantly improved AUC compared with the basic model (all P ≥0.100). The derived-index model had a slightly lower Brier score (0.18) and showed exploratory improvements in continuous NRI (0.16; 95% CI, 0.03-0.29; P = 0.008) and IDI (0.02; 95% CI, 0.01-0.03; P = 0.004). Given the absence of a significant AUC increment and the known limitations of continuous NRI, these reclassification findings were not interpreted as evidence of clinical actionability.

**Table 3:** Repeated nested cross-validated prediction performance of prespecified feature sets for any pulmonary nodule.

Model	Basic risk model	Traditional raw model	Derived-index model	Combined model
No. of unique predictors	11	33	29	50
Events per variable	34.64	11.55	13.14	7.62
AUC (95% CI)	0.76 (0.72-0.78)	0.76 (0.73-0.79)	0.77 (0.74-0.79)	0.76 (0.73-0.79)
AUPRC	0.64	0.66	0.67	0.66
Brier score	0.186	0.183	0.182	0.183
Calibration intercept	-0.02	-0.08	-0.06	-0.11
Calibration slope	1.00	1.06	1.01	1.04
Sensitivity at 0.50	0.45	0.48	0.48	0.50
Specificity at 0.50	0.85	0.84	0.85	0.84
AUC difference vs basic (95% CI)	Reference	0.01 (-0.01, 0.02)	0.01 (-0.00, 0.03)	0.01 (-0.01, 0.02)
P for AUC difference	Reference	0.272	0.100	0.276
Continuous NRI (95% CI)	Reference	0.04 (-0.08, 0.16)	0.16 (0.03, 0.29)	0.04 (-0.08, 0.16)
P for NRI	Reference	0.500	0.008	0.516
IDI (95% CI)	Reference	0.01 (-0.00, 0.02)	0.02 (0.01, 0.03)	0.01 (0.00, 0.03)
P for IDI	Reference	0.096	0.004	0.028

Values are based exclusively on out-of-fold predictions from repeated nested stratified five-fold cross-validation (three repeats), with three-fold inner tuning of ridge regularization. The traditional and derived sets shared 12 variables, so their union contained 33 + 29 - 12 = 50 unique predictors. Paired 95% CIs and P values were obtained by 500 participant-level bootstrap replicates. Continuous NRI and IDI are exploratory and should be interpreted together with discrimination and calibration.

**Figure 4:**
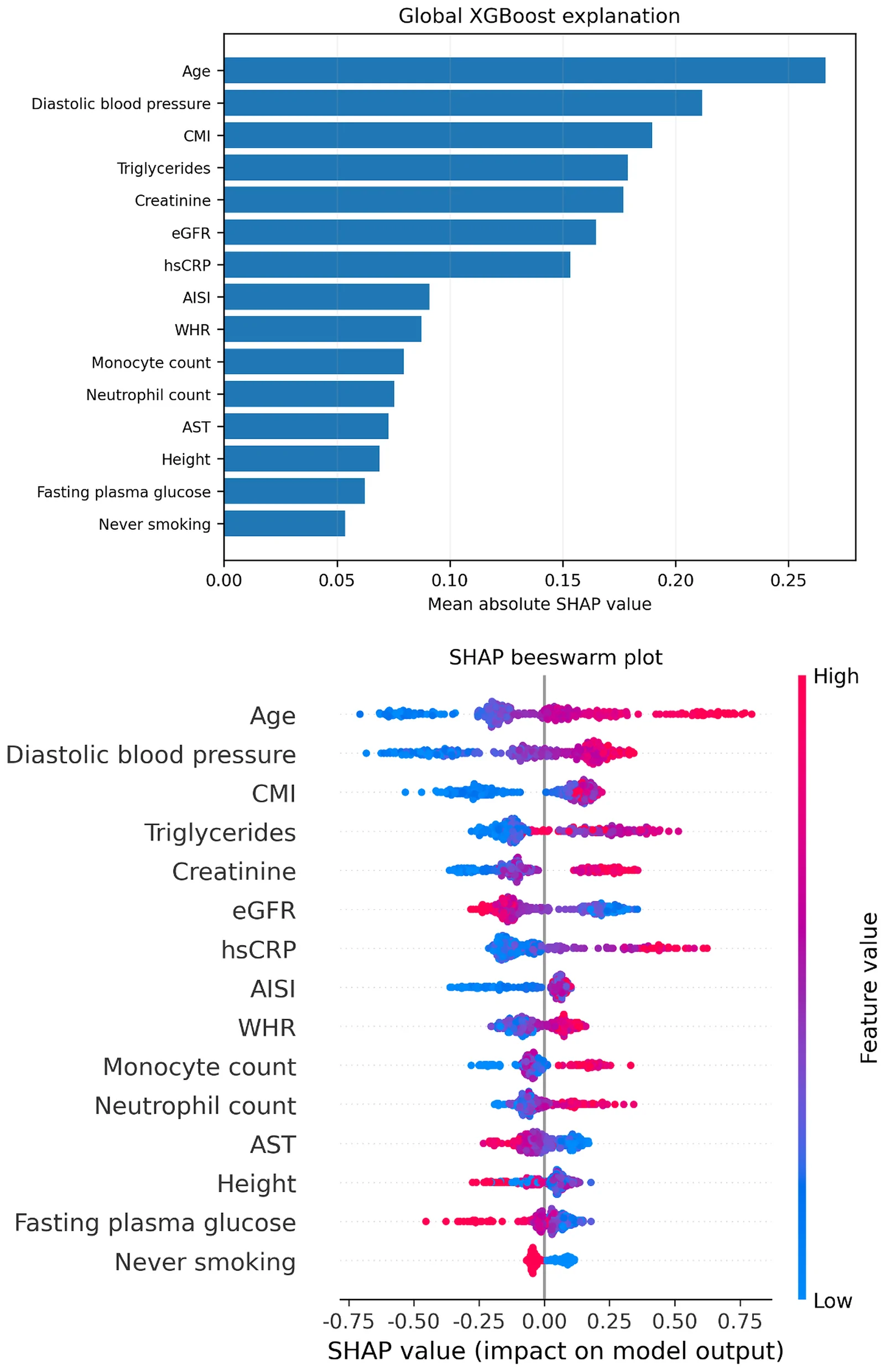
Held-out SHAP explanations for the gradient-boosting model. **(A)** shows pooled mean absolute SHAP importance, and **(B)** shows feature-level SHAP distributions. SHAP values were calculated only for observations in each held-out outer fold and then pooled across folds; they describe model contribution rather than causality.

Across algorithms evaluated with held-out five-fold predictions (**Table 4**), LASSO logistic regression achieved the highest AUC (0.78; 95% CI, 0.75-0.81), with a small AUC difference of 0.009 versus ridge logistic regression (P = 0.004). None of the tree-based models outperformed ridge logistic regression; gradient boosting was the best-performing tree model (AUC, 0.77). SHAP values for gradient boosting were calculated only for held-out outer-fold observations and pooled across folds (**Figure 4**). Age was the most influential feature, followed by CMI and creatinine; SIRI, hsCRP, and eGFR were also among the leading features. Thus, the held-out SHAP pattern contained substantial age- and renal-function information in addition to metabolic and inflammatory signals. These rankings describe predictive model contributions and do not establish independent or causal effects.

**Table 4:** Held-out five-fold cross-validated machine-learning performance for identifying any pulmonary nodule.

Algorithm	Logistic regression	LASSO logistic	Random forest	XGBoost	LightGBM	Gradient boosting
AUC (95% CI)	0.77 (0.74-0.80)	0.78 (0.75-0.81)	0.75 (0.72-0.78)	0.77 (0.73-0.79)	0.77 (0.73-0.79)	0.77 (0.74-0.80)
AUPRC	0.67	0.68	0.64	0.66	0.65	0.65
Sensitivity at 0.50	0.50	0.51	0.40	0.47	0.47	0.42
Specificity at 0.50	0.84	0.85	0.89	0.85	0.84	0.89
Brier score	0.181	0.178	0.188	0.183	0.184	0.184
AUC difference vs logistic (95% CI)	Reference	0.009 (0.003, 0.015)	-0.016 (-0.030, -0.001)	-0.003 (-0.013, 0.010)	-0.002 (-0.016, 0.011)	-0.001 (-0.013, 0.014)
P for AUC difference	Reference	0.004	0.028	0.692	0.816	0.980

All estimates are based on held-out outer-fold predictions. Logistic models used L2 or L1 shrinkage; tree models used conservative depth, minimum-leaf, subsampling, and/or regularization constraints. Paired AUC differences and 95% CIs were obtained by 500 participant-level bootstrap replicates. The small LASSO AUC increment requires external validation.

## 4 Discussion

In this hospital-based cross-sectional health-examination cohort, two findings were most robust after addressing redundancy and multiple testing. In the mutually adjusted representative-index model, AIP and SIRI were independently associated with any CT-detected pulmonary nodule. SIRI was also associated with Lung-RADS category ≥3, but this secondary result arose from a model with only 109 events and an EPV of 6.41 and should therefore be regarded as less statistically stable and hypothesis-generating rather than as a co-equal primary finding. The full exploratory analysis showed related lipid-adiposity and monocyte-driven signals, but the high mathematical overlap among indices means that these should not be counted as numerous independent biomarker discoveries.

The relevance of this work to systems endocrinology lies in the exposures and their cross-organ integration rather than in classifying pulmonary nodules as an endocrine outcome. AIP summarizes atherogenic lipid balance, WHtR reflects adiposity distribution, and inflammatory indices capture systemic immune-cell patterns. Their associations with an extra-endocrine CT phenotype are compatible with coordinated metabolic-inflammatory variation across organ systems. Nevertheless, the present data do not demonstrate an endocrine mechanism, identify a causal pathway, or justify using these indices in place of radiological assessment.

Pulmonary nodules are not a uniform biological entity. A CT-detected nodule may represent a benign inflammatory focus, granuloma, scar, indeterminate lesion, or early malignancy, and the direction and magnitude of biomarker associations may differ across these categories. Pooling all nodules can dilute subtype-specific associations, create an average of opposing patterns, or reflect common factors related to healthcare selection and age rather than a shared disease process. Lung-RADS category ≥3 provides a more standardized higher-attention threshold than an undefined institutional positive flag, but it is not equivalent to pathological malignancy and remains heterogeneous. Moreover, Lung-RADS was developed for lung-cancer screening, whereas the present cohort comprised routine health-examination attendees and was not selected according to guideline-based screening eligibility. The institutional Lung-RADS ≥3 outcome should therefore be interpreted as a pragmatic radiological stratification within this setting, and its risk meaning may not be directly transportable to guideline-defined screening populations.

Monocyte-related indices showed the most consistent associations with Lung-RADS category ≥3. Monocytes and neutrophils participate in systemic inflammatory signaling, tissue remodeling, and tumor-associated immune environments (12–14), and prior studies have linked peripheral inflammatory markers with pulmonary nodule detection or lung cancer risk (9, 44, 45). These observations support biological plausibility, but reverse causation and residual confounding remain possible in a cross-sectional study. The data therefore support an association with an inflammatory phenotype rather than evidence that systemic inflammation caused the nodules.

The lipid-adiposity findings require similar restraint. AIP and CMI share the triglyceride-to-HDL component, while CMI additionally incorporates WHtR; BRI and WHtR are also near-equivalent transformations in this dataset. Consequently, the significant single-index estimates largely describe a smaller number of underlying lipid and anthropometric dimensions. Our revised primary model reduced this redundancy and showed that AIP retained an association with any nodule after simultaneous adjustment, whereas WHtR did not remain significant after FDR correction. The contrast between the exploratory single-index WHtR result (q = 0.004) and the mutually adjusted representative-index result (q = 0.070) is therefore informative: the WHtR association attenuated after the other representative domains were entered simultaneously. Because CMI mathematically combines WHtR with triglyceride-to-HDL information and AIP is itself derived from the triglyceride-to-HDL ratio, the strong single-index WHtR/CMI findings should not be interpreted as evidence that WHtR provides an independent signal. Rather, this pattern is consistent with overlapping metabolic-adiposity information being distributed across correlated composite indices. No representative marker showed evidence of nonlinearity over the central 98% of its distribution.

The prediction results do not establish clinical readiness. Expanded feature sets did not significantly improve AUC, and calibration and Brier-score differences were small. Although the derived-index model improved continuous NRI and IDI, continuous reclassification measures can be unstable or inflated and may indicate movement in predicted probabilities without a meaningful change in discrimination, calibration, or clinical decisions (43). The small AUC advantage of LASSO over ridge logistic regression likewise requires external replication. SHAP analysis was useful for describing held-out model behavior, but feature importance is not equivalent to independent association or causality.

The SHAP results also temper an exclusively metabolic-inflammatory interpretation of the prediction model. Age was the dominant predictor, creatinine ranked above SIRI and hsCRP, and eGFR was also among the leading features. Creatinine and eGFR are overlapping measures of renal function and may additionally capture age-related physiology, comorbidity burden, or other cross-sectional health differences. Their SHAP prominence therefore should not be interpreted as evidence of a direct renal pathophysiological mechanism; residual confounding and reverse causation remain plausible. The different handling of renal variables reflected the distinct goals of the analyses: the association model used eGFR as a single renal-function adjustment covariate to avoid redundant simultaneous adjustment with creatinine, whereas the prediction feature sets allowed both routinely available raw measures to compete as predictors under regularization and/or model constraints. Accordingly, **Figure 4** is better interpreted as a broader age-related systemic prediction pattern containing metabolic, inflammatory, and renal signals, rather than as confirmation of a uniquely metabolic-inflammatory causal axis.

This study has several strengths. It used routinely collected anthropometric, biochemical, and CT-report data from a well-defined health-examination cohort; recalculated all indices from raw components; explicitly separated a reduced representative-index analysis from the full exploratory set; applied FDR control, transformation diagnostics, spline assessment, and repeated out-of-sample evaluation; reported events per model parameter; and calculated SHAP explanations only in held-out folds. Laboratory data were not available to radiologists during interpretation, reducing the likelihood that biomarker values directly influenced radiological reporting.

Several limitations are substantial. First, the cross-sectional design precludes causal or temporal inference. Second, the primary outcome combined heterogeneous pulmonary nodules, and the database did not provide retrievable information on size, density, morphology, location, interval growth, pathology, or longitudinal evolution. Reports were generated by an initial radiologist and reviewed by a second radiologist, but centralized image re-reading was not performed. Underlying lung diseases, including fibrosing interstitial lung disease, can alter both nodule interpretation and longitudinal malignancy risk; recent multimodal longitudinal work illustrates why imaging context and temporal evolution are essential (46). Third, Lung-RADS was developed for lung-cancer screening, whereas this cohort was drawn from routine health examinations and was not selected according to guideline-based screening eligibility. Although ACR Lung-RADS v2022 was used under a standardized institutional process when categorization was requested by a patient or recommended by a physician according to the clinical context, its applicability and risk meaning may differ outside the intended screening context. In addition, Lung-RADS category ≥3 was available only as an institutional binary field; detailed category-specific imaging data were unavailable, and only 109 events (6.41 events per model parameter) limited precision and model stability. Fourth, smoking was captured only as never/former/current; pack-years, cessation duration, occupational and environmental exposures, family history of lung cancer, and detailed chronic lung-disease history were unavailable, leaving important residual confounding. Fifth, examination-defined hypertension was based on a single measurement and did not incorporate prior diagnosis or medication use, creating potential misclassification. Finally, this was a single-center cohort from Shandong without external validation. The findings should therefore be considered exploratory and are not ready for clinical implementation.

Future studies should recruit independent, geographically diverse cohorts and use standardized central radiological adjudication that records nodule size, density, morphology, location, underlying parenchymal disease, and interval change (47). Longitudinal analyses are needed to determine whether metabolic-inflammatory profiles precede nodule development, predict growth or category progression, or merely accompany prevalent findings. Multimodal models should be evaluated against imaging-based standards and judged by calibration, decision consequences, and external transportability rather than reclassification statistics alone.

## 5 Conclusion

In this hospital-based cross-sectional cohort, AIP and SIRI were independently associated with any CT-detected pulmonary nodule. SIRI also showed an exploratory association with Lung-RADS category ≥3, but the limited number of secondary events and EPV of 6.41 require cautious interpretation and independent validation. These findings describe an exploratory metabolic-inflammatory phenotype and do not establish causality, malignancy, or a clinically actionable screening tool. Expanded models did not materially improve discrimination or calibration beyond conventional factors. Multicenter longitudinal validation with standardized radiological characterization and external model assessment is required before any clinical application.

## Data Availability

The original contributions presented in the study are included in the article/**Supplementary Material**. Further inquiries can be directed to the corresponding author.
