# Supplementary material for: Associations of derived inflammatory, lipid, and anthropometric indices with CT-detected pulmonary nodules: a hospital-based cross-sectional study with explainable machine learning
Source: Front Endocrinol (Lausanne). 2026 Sep 1;17:1922394. doi: 10.3389/fendo.2026.1922394 (PMC13574705; doi:10.3389/fendo.2026.1922394)
Supplement: Supplementary file 1 [file Table1.docx]

**Supplementary Tables**

**Supplementary Table S1. Baseline characteristics according to Lung-RADS category ≥3 status.**

| **Variable** | Lung-RADS ≥3 | | | |
| --- | --- | --- | --- | --- |
|  | **Overall** | **No** | **Yes** | **P value** |
| **Age, years** | 48.71 (12.66) | 47.40 (12.23) | 60.21 (10.40) | <0.001 |
| **Height, cm** | 165.74 (7.92) | 165.56 (7.92) | 167.35 (7.79) | 0.025 |
| **Weight, kg** | 66.83 (10.19) | 66.28 (10.10) | 71.77 (9.64) | <0.001 |
| **Waist circumference, cm** | 83.05 (7.27) | 82.58 (7.22) | 87.15 (6.37) | <0.001 |
| **Hip circumference, cm** | 97.27 (5.26) | 97.09 (5.28) | 98.93 (4.88) | <0.001 |
| **Systolic blood pressure, mmHg** | 120.40 (12.49) | 119.69 (12.44) | 126.61 (11.13) | <0.001 |
| **Diastolic blood pressure, mmHg** | 76.34 (7.91) | 75.86 (7.87) | 80.58 (6.93) | <0.001 |
| **White blood cell count, 10^9/L** | 6.26 (0.95) | 6.22 (0.93) | 6.62 (1.04) | <0.001 |
| **Neutrophil count, 10^9/L** | 3.68 (0.68) | 3.65 (0.67) | 3.99 (0.75) | <0.001 |
| **Lymphocyte count, 10^9/L** | 1.96 (0.34) | 1.96 (0.34) | 2.00 (0.34) | 0.249 |
| **Monocyte count, 10^9/L** | 0.41 (0.10) | 0.41 (0.10) | 0.47 (0.12) | <0.001 |
| **Platelet count, 10^9/L** | 236.07 (41.06) | 236.28 (41.58) | 234.23 (36.34) | 0.583 |
| **Hemoglobin, g/L** | 143.78 (12.83) | 143.51 (12.89) | 146.20 (12.06) | 0.030 |
| **Albumin, g/L** | 45.26 (1.98) | 45.38 (1.94) | 44.28 (2.03) | <0.001 |
| **Total cholesterol, mmol/L** | 5.10 (0.85) | 5.04 (0.83) | 5.60 (0.91) | <0.001 |
| **Triglycerides, mmol/L** | 1.68 (0.88) | 1.63 (0.86) | 2.07 (0.96) | <0.001 |
| **HDL-C, mmol/L** | 1.26 (0.21) | 1.27 (0.21) | 1.20 (0.19) | <0.001 |
| **LDL-C, mmol/L** | 2.91 (0.63) | 2.87 (0.61) | 3.30 (0.67) | <0.001 |
| **Fasting plasma glucose, mmol/L** | 5.31 (0.77) | 5.27 (0.75) | 5.64 (0.80) | <0.001 |
| **ALT, U/L** | 27.66 (10.66) | 27.22 (10.56) | 31.62 (10.73) | <0.001 |
| **AST, U/L** | 23.04 (6.45) | 22.99 (6.38) | 23.52 (7.00) | 0.445 |
| **GGT, U/L** | 37.14 (21.50) | 36.76 (21.20) | 40.47 (23.86) | 0.122 |
| **Creatinine, umol/L** | 71.34 (12.47) | 70.84 (12.47) | 75.83 (11.53) | <0.001 |
| **eGFR, mL/min/1.73 m^2** | 105.84 (11.20) | 106.67 (11.03) | 98.42 (9.91) | <0.001 |
| **Uric acid, umol/L** | 334.69 (67.68) | 330.87 (67.19) | 368.51 (62.71) | <0.001 |
| **hsCRP, mg/L** | 1.42 (1.19) | 1.34 (1.07) | 2.16 (1.79) | <0.001 |
| **BMI, kg/m^2** | 24.25 (2.66) | 24.10 (2.66) | 25.56 (2.33) | <0.001 |
| **WHtR** | 0.50 (0.04) | 0.50 (0.04) | 0.52 (0.04) | <0.001 |
| **WHR** | 0.85 (0.07) | 0.85 (0.07) | 0.88 (0.06) | <0.001 |
| **Non-HDL-C, mmol/L** | 3.84 (0.88) | 3.77 (0.85) | 4.39 (0.97) | <0.001 |
| **Remnant cholesterol, mmol/L** | 0.93 (0.43) | 0.91 (0.43) | 1.10 (0.45) | <0.001 |
| **AIP** | 0.08 (0.25) | 0.06 (0.25) | 0.20 (0.23) | <0.001 |
| **CMI** | 0.71 (0.47) | 0.69 (0.44) | 0.96 (0.62) | <0.001 |
| **WWI** | 10.20 (0.67) | 10.19 (0.68) | 10.33 (0.63) | 0.032 |
| **BRI** | 3.42 (0.81) | 3.38 (0.81) | 3.80 (0.75) | <0.001 |
| **NLR** | 1.89 (0.29) | 1.88 (0.28) | 2.01 (0.31) | <0.001 |
| **PLR** | 124.17 (32.52) | 124.56 (32.86) | 120.81 (29.29) | 0.214 |
| **MLR** | 0.21 (0.05) | 0.21 (0.05) | 0.24 (0.05) | <0.001 |
| **SII** | 447.42 (106.61) | 444.89 (107.13) | 469.78 (99.51) | 0.015 |
| **SIRI** | 0.79 (0.27) | 0.77 (0.26) | 0.96 (0.32) | <0.001 |
| **AISI** | 187.18 (74.44) | 183.21 (72.96) | 222.27 (78.44) | <0.001 |
| **MHR** | 0.34 (0.12) | 0.33 (0.11) | 0.40 (0.13) | <0.001 |
| **PNI** | 55.08 (2.64) | 55.17 (2.62) | 54.27 (2.73) | 0.001 |
| **HALP** | 56.15 (16.23) | 56.04 (16.19) | 57.18 (16.69) | 0.497 |
| **Sex** |  |  |  | 0.002 |
| Male | 596 (55.60) | 520 (54.00) | 76 (69.72) |  |
| Female | 476 (44.40) | 443 (46.00) | 33 (30.28) |  |
| **Smoking status** |  |  |  | <0.001 |
| Never | 708 (66.04) | 661 (68.64) | 47 (43.12) |  |
| Former | 83 (7.74) | 69 (7.17) | 14 (12.84) |  |
| Current | 281 (26.21) | 233 (24.20) | 48 (44.04) |  |
| **Current alcohol drinking** |  |  |  | 0.024 |
| No | 790 (73.69) | 720 (74.77) | 70 (64.22) |  |
| Yes | 282 (26.31) | 243 (25.23) | 39 (35.78) |  |
| **Regular exercise** |  |  |  | 0.895 |
| No | 621 (57.93) | 559 (58.05) | 62 (56.88) |  |
| Yes | 451 (42.07) | 404 (41.95) | 47 (43.12) |  |
| **Central obesity** |  |  |  | 0.006 |
| No | 702 (65.49) | 644 (66.87) | 58 (53.21) |  |
| Yes | 370 (34.51) | 319 (33.13) | 51 (46.79) |  |
| Examination-defined hypertension |  |  |  | <0.001 |
| No | 967 (90.21) | 882 (91.59) | 85 (77.98) |  |
| Yes | 105 (9.79) | 81 (8.41) | 24 (22.02) |  |

*Data are shown as mean (SD) or No. (%). For categorical variables, levels are shown as indented rows and the global P value is presented on the parent variable row. P values were calculated using Welch t tests or chi-square tests, as appropriate. P values are formatted as <0.001 or to three decimal places. Lung-RADS category ≥3 was the prespecified secondary outcome. Central obesity was defined as waist circumference ≥90 cm in men or ≥80 cm in women. Examination-defined hypertension was based on a single measurement with systolic blood pressure ≥140 mmHg or diastolic blood pressure ≥90 mmHg.*

**Supplementary Table S2. FDR-controlled exploratory single-index associations with any pulmonary nodule and Lung-RADS category ≥3.**

| **Index** | **Any pulmonary nodule OR (95% CI)** | **P value** | **FDR q value** | **Lung-RADS ≥3 OR (95% CI)** | **P value** | **FDR q value** |
| --- | --- | --- | --- | --- | --- | --- |
| BMI | 1.25 (1.05-1.49) | 0.012 | 0.030 | 1.31 (0.99-1.72) | 0.055 | 0.095 |
| Waist circumference | 1.36 (1.12-1.64) | 0.002 | 0.005 | 1.30 (0.97-1.75) | 0.077 | 0.128 |
| WHtR | 1.32 (1.12-1.55) | <0.001 | 0.004 | 1.18 (0.91-1.51) | 0.206 | 0.279 |
| WHR | 1.21 (1.01-1.45) | 0.042 | 0.080 | 1.12 (0.85-1.48) | 0.405 | 0.491 |
| Non-HDL-C | 1.23 (1.02-1.47) | 0.029 | 0.061 | 1.06 (0.81-1.39) | 0.669 | 0.724 |
| Remnant cholesterol | 1.22 (1.04-1.43) | 0.014 | 0.033 | 0.98 (0.78-1.23) | 0.838 | 0.882 |
| AIP | 1.41 (1.18-1.69) | <0.001 | 0.001 | 1.08 (0.83-1.42) | 0.561 | 0.660 |
| CMI (ln) | 1.52 (1.27-1.81) | <0.001 | <0.001 | 1.20 (0.92-1.56) | 0.184 | 0.263 |
| WWI | 1.18 (1.02-1.36) | 0.031 | 0.061 | 1.01 (0.81-1.27) | 0.929 | 0.929 |
| BRI | 1.32 (1.12-1.55) | <0.001 | 0.004 | 1.16 (0.91-1.48) | 0.225 | 0.282 |
| NLR | 1.25 (1.06-1.46) | 0.006 | 0.017 | 1.16 (0.92-1.47) | 0.217 | 0.279 |
| PLR | 1.11 (0.96-1.28) | 0.172 | 0.255 | 0.99 (0.78-1.25) | 0.904 | 0.928 |
| MLR | 1.39 (1.19-1.63) | <0.001 | <0.001 | 1.50 (1.19-1.89) | <0.001 | 0.004 |
| SII | 1.23 (1.06-1.42) | 0.006 | 0.017 | 1.06 (0.84-1.32) | 0.634 | 0.705 |
| SIRI | 1.44 (1.21-1.71) | <0.001 | <0.001 | 1.49 (1.17-1.90) | 0.001 | 0.005 |
| AISI (ln) | 1.40 (1.18-1.65) | <0.001 | <0.001 | 1.36 (1.05-1.76) | 0.020 | 0.045 |
| MHR | 1.41 (1.18-1.69) | <0.001 | 0.001 | 1.49 (1.16-1.92) | 0.002 | 0.007 |
| PNI | 0.89 (0.77-1.03) | 0.125 | 0.193 | 0.83 (0.66-1.05) | 0.122 | 0.193 |
| HALP | 0.86 (0.73-1.00) | 0.051 | 0.092 | 0.94 (0.74-1.20) | 0.619 | 0.705 |
| hsCRP (ln) | 1.29 (1.10-1.52) | 0.002 | 0.007 | 1.17 (0.91-1.49) | 0.216 | 0.279 |

*ORs are per 1-SD increment. Each index was modeled separately with adjustment for age, sex, smoking status, current alcohol drinking, regular exercise, examination-defined hypertension, fasting plasma glucose, uric acid, eGFR, ALT, and GGT; BMI was additionally included when the index was not adiposity-derived. CMI, AISI, and hsCRP were natural-log transformed before standardization. Benjamini-Hochberg q values were calculated jointly across all 40 tests. These results are exploratory because several indices share mathematical components. Examination-defined hypertension was based on a single measurement with systolic blood pressure ≥140 mmHg or diastolic blood pressure ≥90 mmHg.*

**Supplementary Table S3. Exploratory subgroup analyses of representative indices for any pulmonary nodule.**

| **Index** | **Subgroup** | **Level** | **OR (95% CI)** | **P value** | **P for interaction** |
| --- | --- | --- | --- | --- | --- |
| SIRI | Sex | Male | 1.43 (1.16-1.76) | <0.001 | 0.519 |
| SIRI | Sex | Female | 1.55 (1.13-2.14) | 0.007 | 0.519 |
| SIRI | Age | <50 years | 1.32 (1.02-1.71) | 0.038 | 0.842 |
| SIRI | Age | ≥50 years | 1.47 (1.17-1.84) | <0.001 | 0.842 |
| SIRI | Central obesity | No | 1.48 (1.20-1.83) | <0.001 | 0.482 |
| SIRI | Central obesity | Yes | 1.46 (1.07-1.99) | 0.016 | 0.482 |
| SIRI | Hypertension | No | 1.37 (1.14-1.64) | <0.001 | 0.142 |
| SIRI | Hypertension | Yes | 2.26 (1.10-4.68) | 0.027 | 0.142 |
| AIP | Sex | Male | 1.49 (1.18-1.87) | <0.001 | 0.783 |
| AIP | Sex | Female | 1.37 (1.02-1.86) | 0.038 | 0.783 |
| AIP | Age | <50 years | 1.42 (1.08-1.87) | 0.011 | 0.825 |
| AIP | Age | ≥50 years | 1.52 (1.20-1.93) | <0.001 | 0.825 |
| AIP | Central obesity | No | 1.40 (1.12-1.77) | 0.004 | 0.715 |
| AIP | Central obesity | Yes | 1.43 (1.05-1.93) | 0.023 | 0.715 |
| AIP | Hypertension | No | 1.41 (1.16-1.71) | <0.001 | 0.468 |
| AIP | Hypertension | Yes | 1.34 (0.71-2.54) | 0.363 | 0.468 |
| WHtR | Sex | Male | 1.38 (1.11-1.73) | 0.004 | 0.660 |
| WHtR | Sex | Female | 1.28 (1.00-1.64) | 0.051 | 0.660 |
| WHtR | Age | <50 years | 1.22 (0.95-1.56) | 0.117 | 0.477 |
| WHtR | Age | ≥50 years | 1.49 (1.20-1.84) | <0.001 | 0.477 |
| WHtR | Central obesity | No | 1.16 (0.88-1.52) | 0.304 | 0.439 |
| WHtR | Central obesity | Yes | 1.36 (0.92-2.00) | 0.120 | 0.439 |
| WHtR | Hypertension | No | 1.29 (1.09-1.53) | 0.004 | 0.994 |
| WHtR | Hypertension | Yes | 1.57 (0.80-3.07) | 0.192 | 0.994 |

*ORs are per 1-SD increment. Interaction P values were obtained from cross-product terms. Subgroup analyses were prespecified but exploratory and were not additionally adjusted for multiple testing. Central obesity was defined as waist circumference ≥90 cm in men or ≥80 cm in women. Examination-defined hypertension was based on a single measurement with systolic blood pressure ≥140 mmHg or diastolic blood pressure ≥90 mmHg.*

**Supplementary Table S4. Distribution diagnostics and transformation procedures for examined indices.**

| **Index** | **Skewness** | **Transformation before standardization** |
| --- | --- | --- |
| BMI | -0.06 | None |
| Waist circumference | -0.16 | None |
| WHtR | -0.02 | None |
| WHR | 0.02 | None |
| Non-HDL-C | 0.28 | None |
| Remnant cholesterol | 0.96 | None |
| AIP | 0.06 | Published log10 ratio retained |
| CMI | 2.08 | Natural logarithm |
| WWI | 0.14 | None |
| BRI | 0.22 | None |
| NLR | 0.77 | None |
| PLR | 0.89 | None |
| MLR | 0.66 | None |
| SII | 0.58 | None |
| SIRI | 0.92 | None |
| AISI | 1.16 | Natural logarithm |
| MHR | 0.82 | None |
| PNI | 0.02 | None |
| HALP | 0.72 | None |
| hsCRP | 2.33 | Natural logarithm |

*Skewness was calculated without reference to outcome status. Positive variables with skewness >1.0 were natural-log transformed. AIP was already defined as log10(triglycerides/HDL-C) and was therefore not transformed again.*
